# Joint optimization of green vehicle scheduling and routing problem with time-varying speeds

**DOI:** 10.1371/journal.pone.0192000

**Published:** 2018-02-21

**Authors:** Dezhi Zhang, Xin Wang, Shuangyan Li, Nan Ni, Zhuo Zhang

**Affiliations:** 1 School of Traffic & Transportation Engineering, Central South University, Changsha, Hunan, China; 2 National & Local Joint Engineering Research Center of Safety Technology for Rail Vehicle, Changsha, Hunan, China; 3 Key Laboratory of Traffic Safety on Track (Central South University), Ministry of Education, Changsha, Hunan, China; 4 School of Logistics and Transportation, Central South University of Forestry and Technology, Changsha, Hunan, China; 5 Joint International Research Laboratory of Key Technology for Rail Traffic Safety, Changsha, Hunan, China; 6 Smart Transport Key Laboratory of Hunan Province, Changsha, Hunan, China; Beihang University, CHINA

## Abstract

Based on an analysis of the congestion effect and changes in the speed of vehicle flow during morning and evening peaks in a large- or medium-sized city, the piecewise function is used to capture the rules of the time-varying speed of vehicles, which are very important in modelling their fuel consumption and CO_2_ emission. A joint optimization model of the green vehicle scheduling and routing problem with time-varying speeds is presented in this study. Extra wages during nonworking periods and soft time-window constraints are considered. A heuristic algorithm based on the adaptive large neighborhood search algorithm is also presented. Finally, a numerical simulation example is provided to illustrate the optimization model and its algorithm. Results show that, (1) the shortest route is not necessarily the route that consumes the least energy, (2) the departure time influences the vehicle fuel consumption and CO_2_ emissions and the optimal departure time saves on fuel consumption and reduces CO_2_ emissions by up to 5.4%, and (3) extra driver wages have significant effects on routing and departure time slot decisions.

## Introduction

Environmental issues have attracted considerable public attention around the world. A wide consensus holds that the transportation system is a major contributor to climate change and global warming. Studies have shown that freight transportation contributes to approximately 5.5% of global greenhouse gas (GHG) emissions [1]. The report on CO_2_ emissions in 25 European countries during the period 1990–2005 also indicated that approximately 26% of the total CO_2_ emissions in the air come from the transportation system. In logistics service activities, CO_2_ emissions from the transportation system were found to account for 93% of the total pollutant emissions, whereas those from warehousing account for only 7% [2]. Thus, creating an environmentally sustainable logistics system is necessary.

Among the logistics activities, the vehicle routing problem (VRP) is one of mostly widely researched, which was first introduced by Dantzig and Ramser [3]. The traditional VRP aims to minimize the total cost or total distance travelled by a fleet of vehicles that start from and end at one depot. Its variants, such as the multi-depot vehicle routing problem, open vehicle routing problem, vehicle routing problem with time-windows, and dynamic vehicle routing problem, have been the subjects of considerable research in the past years, mostly because of the additional constraints and objectives arising from real-world applications[4,5].

Drexl [6] compared the traditional VRP and its variants. Lysgaard and Wøhlk [7] proposed an exact algorithm for the cumulative capacitated vehicle routing problem to minimize the total arrival time rather than the total distance or time travelled.

A growing body of research has pointed out a concern regarding the environment; thus, new vehicle routing models have been presented to account for not only the travel costs or distances but also the energy consumption and GHG emissions [8–14]. İ. Kara et al. [8] proposed the energy-minimizing VRP to reduce the total energy consumed. Kwon et al. [9] studied a VRP, whose objective is to minimize the total operational cost of heterogeneous vehicles, including CO_2_ emissions cost. Palmer [15] investigated an integrated routing and CO_2_ emission model for goods vehicles and developed a model that was tested on a case study of home deliveries for grocery stores in the United Kingdom. The author found that an average saving of 4.8% in CO_2_ emissions is possible compared to using routes that minimize time, but at the expense of a 3.8% increase in the required time. Bektaş and Laporte [11] proposed the pollution-routing problem, a new variant of VRP, to minimize the total cost, which includes the cost of fuel consumption, the corresponding GHG emission cost, and driver wages. Zhu et al. [16] proposed a procedure to find the maximum-capacity path while considering both fuel consumption and total cost.

Recent research on minimizing emissions in vehicle routing models can be divided into two main categories: (1) the set of models where time independence is assumed and (2) the set of models where the road conditions are subject to traffic congestion, such that the time needed to travel along a road segment depends on the departure time of vehicles [4].

The fuel consumption and CO_2_ emissions of delivery vehicles are closely related to the driving speed, load and road conditions, and other factors. Most researchers define vehicle speed as a constant variable. Some Researchers have conducted surveys that revealed that fuel cost accounts for a large proportion of transportation costs [17]. According to a report, fuel cost accounts for over 46% of the operational cost of a logistics company in China [18]. Figliozzi [19] studied CO_2_ emissions on different road conditions and time-definite customer demands. The result indicated that road conditions and speed limits have significant influences on CO_2_ emissions. By implementing several congestion mitigation techniques, CO_2_ emissions can be reduced by up to 20% [20]. Demir et al. [21] studied the VRP with CO_2_ emissions and proposed a corresponding optimization model and solution algorithm. Pradenas et al. [22] added return transportation to the VRP, thereby avoiding the return of the vehicle load to mitigate GHG emissions.

Van Woensel et al. [23] compared a queuing approach with other approaches to solve a VRP with time-varying speed due to different road conditions. Maden et al. [24] studied the VRP with time-varying speed and found that fuel consumption and emissions can be reduced by 7% using the vehicle route optimization with real-time traffic congestion information on the road network. Ropke and Pisinger [25] proposed an adaptive large neighborhood search (ALNS) algorithm to deal with pickup and delivery problems with time windows and found that the ALNS algorithm is capable of finding solutions of good quality in a reasonable time. Lorini et al. [26] proposed a dynamic VRP model where travel times and demands are assumed to be dynamic. Figliozzi [27] analyzed the computational complexity of the genetic algorithm (GA), tabu search (TS), and ant colony algorithm in solving time-dependent VRP (TDVRP). Jabali et al. [28] proposed a model that considers CO_2_ emission-related costs, including fuel consumption, travel time, and emissions that are time-dependent, and the model is solved using a TS procedure. Franceschetti et al. [29] described the pollution-routing problem in time-dependent environment using an integer linear programming. Wen et al. [30] studied the optimal path selection problem along with the road congestion pricing and road time-varying speed conditions. Wang, Y. et al. [31] addressed a collaborative two-echelon logistics joint distribution network design problem, which is solved by a hybrid algorithm integrated an ant colony optimization algorithm and genetic algorithm. Taş et al. [32] studied a TDVRP with stochastic travel times and proposed two meta-heuristics: TS and ALNS. Ehmke et al. [33] focused on minimizing the CO_2_ emissions in urban areas while considering the effect of travelling at traffic speed and vehicle loads and proposed a technique to reduce computational difficulties. Wen and Eglese [34] investigated the minimum-cost VRP with time-dependent speed data and congestion charge scheme. They introduced a heuristic algorithm called LANCOST to minimize the total travel cost, which includes fuel cost, driver cost, and congestion charge. Luo et al. [35] applied the ALNS heuristics and dynamic programming recursions to the VRP, which considers stochastic demands and weight-related cost. Qian and Eglese [4] addressed the optimization of fuel emissions in VRPs with time-varying speeds, considering the capacities of the vehicles and the time constraints on the total length of each route. They presented a column generation based on the TS to solve the problem. Xiao and Konak [36] addressed a TDVRP with the objective of minimizing the emitted CO_2_ and proposed a hybrid solution approach that combines a GA with an exact dynamic programming procedure. Xiao and Konak [37] proposed a hybrid algorithm of mixed-integer linear programming and iterated neighborhood search to deal with the green vehicle scheduling and routing problem with time windows, which considers time-dependent road conditions. Huang et al. [38] considered a TDVRP with path selection and formulated models on both stochastic and deterministic conditions.

A road timetable was designed to provide the expected times for vehicles to travel between locations starting at different times [39]. In a TDVRP, the first in, first out method is an important property, and the time-varying speed is defined as a constant at a certain period to guarantee this property [40].

In this study, we consider the time window for each customer. Although most researchers consider time windows as hard constraints, some practical applications have shown that time-window constraint can be violated at a cost [41].

To the best of our knowledge, existing related studies integrating vehicle scheduling and routing problem considering different wages in different time slots are still scarce. In this study, we focus on the optimization of minimizing the total cost, which includes fuel consumption, different driver wages in different time slots, and CO_2_ emissions, in time-varying road conditions. Solving the problem is immensely time-consuming, thus an ALNS algorithm is proposed. The main contributions of this study are twofold. First, a joint optimization model of the departure time schedule of vehicles and the routing problem with time-varying speeds is presented, and CO_2_ emissions and soft time-window constraints are considered. Second, higher wages for drivers who are working during nonworking periods are considered, and the trade-off between the total cost and CO_2_ emissions is analyzed.

The rest of this paper is organized as follows. Section 2 describes the green vehicle scheduling and routing problem with time-varying speeds, whose objective is to minimize the total costs, which include fuel cost, CO_2_ emissions, time-window violation penalty, and driver wages. Section 3 introduces the framework of the ALNS algorithm. Section 4 presents a case study to examine the efficiency of the proposed algorithm and the managerial insights are obtained according to the results. Finally, Section 5 presents the conclusions and recommendations for further studies.

## Problem analysis and modelling

### Problem description

**Fig 1:**
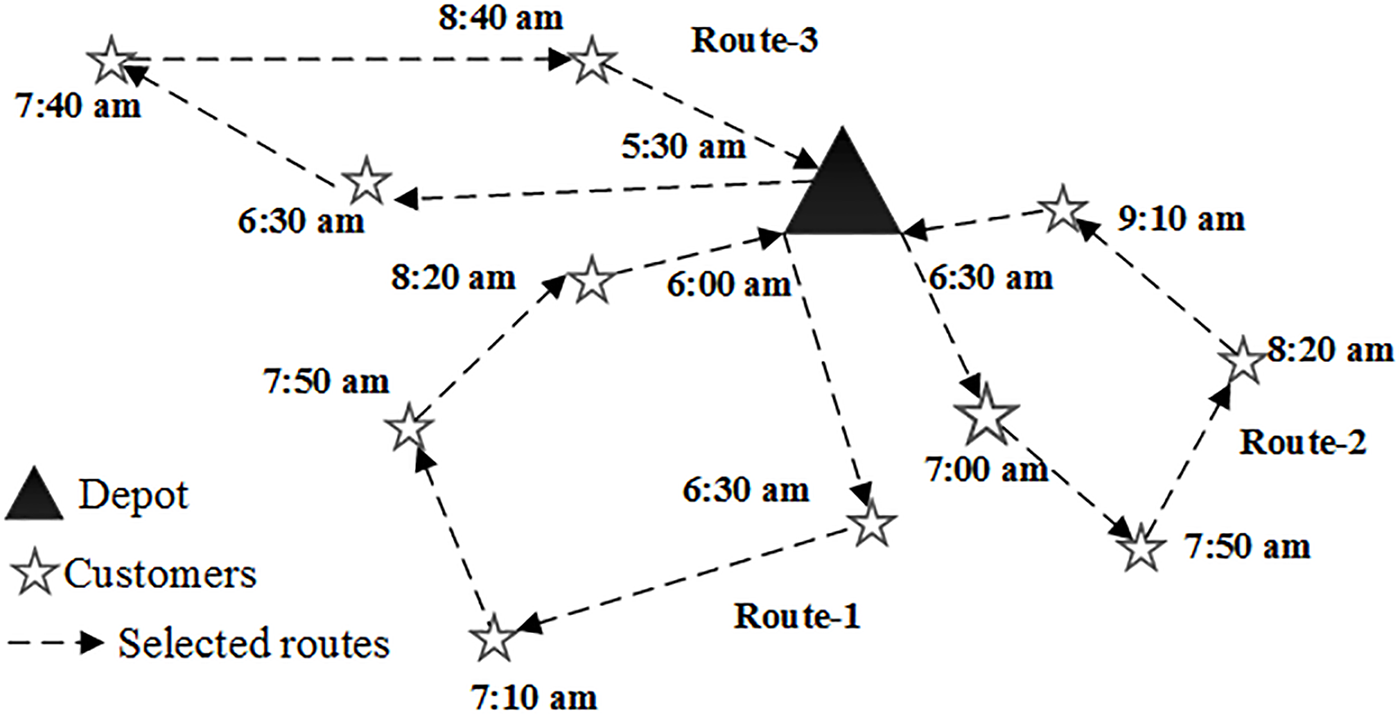
A diagram of the solution of joint vehicle scheduling and routing problem.

The general assumption in the traditional vehicle routing problem is that vehicle speed is constant, and few considerations reflect the actual road conditions. Vehicle speed cannot be constant all the time and is in fact constantly changing due to different road conditions. Thus, our study mainly focuses on the joint optimization of the vehicle scheduling and routing problem with time-varying speeds, CO_2_ emission considerations, and higher wages during nonworking periods. The fuel consumption and CO_2_ emissions of a vehicle are undoubtedly associated with the driving speed and load weight. We consider the fixed cost, the delivery cost, the time-window violation cost, and the external cost of vehicle emissions. We also study the optimization problem of the routes and the schedules of a fleet of delivery vehicles that minimize the fuel emissions in a road network where speed depends on time. A brief demonstration of the solution is presented in Fig 1.

### Hypotheses

A1: The location of a warehouse (i.e. depot) and the customers are known.

A2: The time-window constraint can be violated at a cost.

A3: The demand and service time windows of each customer are known.

A4: Orders cannot be split, that is, each customer has only one vehicle for its services.

A5: Only one type of vehicle is available, and its dead weight and load capacity are 2.5 and 7 tons, respectively. The number of vehicles is sufficient.

A6: In the urban traffic network, the change rule of average vehicle speed is ladder-like.

A7: The per-unit-time reward for drivers during the nonworking period (i.e., earlier than 8:00 a.m. or later than 6:00 p.m.) is higher than that during the normal work period (between 8:00 a.m. and 8:00 p.m.).

**Fig 2:**
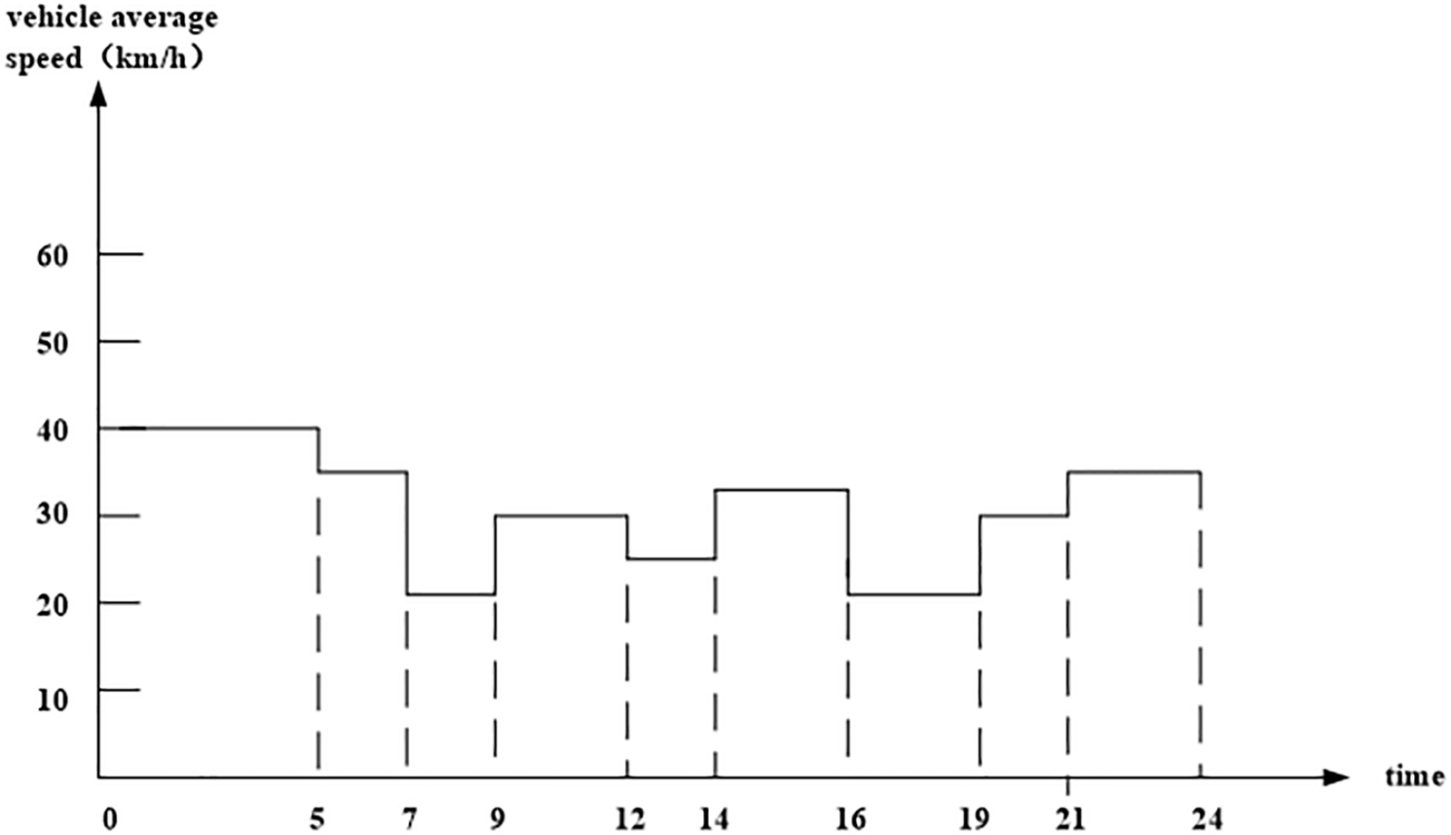
Vehicle average speed change curve on roads of Changsha City. (Source: Data adapted from the references [42,43]).

In the urban traffic network, congestion must be considered. Congestion often occurs from 7:00 a.m. to 9:00 a.m., from12:00 p.m. to 1:00 p.m., and from 6:00 p.m. to 7:00 p.m. The data and change rule of speed extracted from the “Changsha Traffic Status Report 2012” in Changsha City, Hunan Province are illustrated in Fig 2.

The fuel consumption of vehicles when considering time-varying speeds is calculated using Eq (1).

In a given arc (*i*, *j*), the vehicle speed is *v*, the total vehicle weight (including the load weight) is *f*, and the arc length is *d*. The fuel consumption of the vehicle can then be calculated using the formulation proposed by Demir et al. [10]:

$$F\left(f,v,d\right)=0.0308d\left(33/v+0.8175+0.2725f+0.0035v^{2}\right)$$ (1)

### Mathematical model

#### Notations

Let *G* = (*N*, *A*) be a customer service network, where *N* = {0,1,…,n} is a set of nodes, *n* = 0 is a warehouse, *N*_0_ = {1,…,*n*} is a set of customer nodes, *A* = {(*i*, *j*)∈*N* × *N*} is a set of arcs, *D* = {*d*_*ij*_|(*i*, *j*)∈*A*} is a set of arc lengths, and *K* = {1,…,*k*} is a set of homogeneous vehicles. The following are other symbols:

$$N\prime =N\cup \left\{n+1\right\},\;N_{0}\prime =N\prime \backslash \left\{0,n+1\right\}$$

$$A^{\prime }=\left\{\left(i,j\right):i,j\in N\prime \right\}$$

$$\begin{array}{ll} x_{ij}^{k}= & \{\begin{array}{l} 1,\;\text{the}\;\text{vehicle}\;k\;\text{travels}\;\text{on}\;\text{arc}\;(i,j)\\ 0,\;\text{else}\; \end{array} \end{array}$$

*w* weight of vehicle

*Q* load capacity of vehicle

*f*_*ij*_ current load of vehicle travels on (*i*, *j*)

*F*_*ij*_ fuel consumption of the vehicle on (*i*, *j*)

*q*_*i*_ demand of customer *i*

*a*_*i*_ earliest arrival time of customer *i*

*b*_*i*_ latest arrival time of customer *i*

$y_{i}^{k}$ arrival time of vehicle k to customer *i*

*ts*^1^ earliest time for vehicles to leave the depot

*ts*^*M*+1^ latest time for vehicles to return to the depot

*m*^*k*^ starting time for the *k-*th vehicle at the depot

*t*_*i*_ service time at customer *i*

*r*_*i*_ departure time at customer *i*

*c*_*f*_ unit cost of the fuel

*c*_*e*_ unit CO_2_ emission cost

*f*_*p*_ CO_2_ emission factor

*c*_*w*_ per-unit-time reward for drivers during normal work period

*c*_*a*_ per-unit-time reward for drivers during abnormal work period

*c*_*k*_ fixed use cost of vehicle *k*

*tv*^*i*^ violated time to serve customer *i*

For practical reasons, we divide a day into several time slots based on the rule of speed variation, that is, M time slots. *T*_1_, *T*_2_,…,*T*_*M*_ denote the first, second, …, and *m*-th slot, and the start and end times of the *m*-th time slot are marked by [*ts*^*m*^, *ts*^*m*+1^], respectively. During the same slot, speed *s*_*m*_ is constant.

In time-varying traffic network, the travel times are time-dependent, i.e., each arc (*i*, *j*) has a dynamic travel time *t*_*ij*_ depending on departure time slot from node *i*. Thus, the traveling time of the vehicle over the arc (*i*, *j*) is dynamic and depends on departure time *r*_*i*_. If the vehicle visits arc (*i*, *j*) using the successive *p+1* time slots, then it has a corresponding *p*+1 types of travel speeds over the trip from customer *i* to customer *j*. If the vehicle leaves customer *i* during the *m*-th period, then the speed of the vehicle leaving customer *i* can be marked as $v_{ij}^{m}(r_{i})$, and the following time period can be marked as $v_{ij}^{m\text{+1}}(r_{i})$. Similarly, the speed at which the vehicle eventually leaves arc (*i*, *j*) is $v_{ij}^{m+p}(r_{i})$, and the corresponding traveling distance is $d_{ij}^{m+p}(r_{i})$.

In general, the speed of the vehicle on arc (*i*, *j*) can be estimated as

$$v_{{}_{ij}}\left(r_{i}\right)=\left\{v_{ij}^{m}\left(r_{i}\right),v_{ij}^{m+1}\left(r_{i}\right),\ldots ,v_{ij}^{m+p}\left(r_{i}\right)\right\}$$ (2)

In arc (*i*, *j*), the traveling time of the vehicle can be expressed as a function related to the departure time when vehicle k leaves node *i*:

$$\tau_{ij}\left(r_{i}\right)=d_{ij}^{m}\left(r_{i}\right)/v_{ij}^{m}\left(r_{i}\right)+d_{ij}^{m+1}\left(r_{i}\right)/v_{ij}^{m+1}\left(r_{i}\right)+\ldots +d_{ij}^{m+p}\left(r_{i}\right)/v_{ij}^{m+p}\left(r_{i}\right)$$ (3)

The energy consumption of the vehicle in arc (*i*, *j*) can be written as

$$F_{ij}\left(w+f_{ij},{\bar{v}}_{ij},d_{ij}\right)=\sum_{l=0}^{p}F_{ij}\left(w+f_{ij},v_{ij}^{m+1},d_{ij}^{m+1}\right)$$ (4)

In Eq (3), we let *v*_*ij*_ denote the average traveling speed of the vehicle over arc (*i*, *j*). The total vehicle weight equals to (*w* + *f*_*ij*_), where w is the vehicle weight and *f*_*ij*_ is the load of the vehicle on arc (*i*, *j*).

To calculate the cost of time-window violation penalty for all customers, the arrival and departure times on customer *i* of vehicle *y*_*i*_ and *r*_*i*_ are needed. [*a*_*i*_, *b*_*i*_] is the time window of customer *i*. The violated time to serve customer *i* is calculated as follows:

if *y*_*i*_ < *a*_*i*_:

$$t_{v}^{i}=a_{i}-y_{i}$$ (5)

if *y*_*i*_ > *b*_*i*_:

$$t_{v}^{i}=y_{i}-b_{i}$$ (6)

In other situations, the time window of customer *i* is not violated and no extra costs are paid.

The energy consumption also changes because of the change of speed. The optimization objectives of this study are shown as follows: to determine the optimal visiting route for each vehicle and to choose the optimal departure time for each vehicle from several available time slots. During the calculation process, vehicle routing and departure times are simultaneously optimized while considering the energy consumption. The objective function (7) includes five parts, namely, fixed cost, driver wages, penalty cost of time-window violation, cost of fuel consumption, and cost of CO_2_ emissions.

$$\begin{array}{l} \text{min}z=\sum_{k\in K}\sum_{j\in N_{0}^{\prime }}c_{k}x_{0j}^{k}+f\left(c_{w},c_{a},y_{n+1}^{k},y_{0}^{k}\right)+\sum_{k\in K}\sum_{\left(i,j\right)\in A\prime }c_{f}F_{ij}\left(w+f_{ij},{\bar{v}}_{ij},d_{ij}\right)\\ +\sum_{i\in N_{0}^{\prime }}c_{v}t_{v}^{i}+\sum_{k\in K}\sum_{\left(i,j\right)\in A\prime }c_{e}f_{p}F_{ij}\left(w+f_{ij},{\bar{v}}_{ij},d_{ij}\right) \end{array}$$ (7)

to:

$$\sum_{j\in N^{\prime }}x_{0j}^{k}=1\;\forall k\in K$$ (8)

$$\sum_{j\in N_{}{}^{\prime }}x_{j,n+1}^{k}=1\;\forall k\in K$$ (9)

$$\sum_{k\in K}\sum_{j\in N^{\prime }}x_{ij}^{k}=1\;\forall i\in N_{0}^{\prime }$$ (10)

$$\sum_{i\in N^{\prime }}x_{il}^{k}-\sum_{j\in N^{\prime }}x_{lj}^{k}=0\;\forall l\in N_{0}^{\prime },k\in K$$ (11)

$$\sum_{i\in N_{0}^{\prime }}q_{i}\sum_{j\in N\prime }x_{lj}^{k}\leq Q\;\forall k\in K$$ (12)

$$G\left(c_{w},c_{a},y_{n+1}^{k},y_{0}^{k}\right)=\left\{ \begin{array}{l} \sum_{k\in K}c_{w}\left(y_{n+1}^{k}-8\right)+c_{a}\left(8-y_{0}^{k}\right),\;\text{if}\;y_{0}^{k}\leq 8\leq y_{n+1}^{k}\leq 18\\ \sum_{k\in K}c_{w}\left(y_{n+1}^{k}-y_{0}^{k}\right),\;\text{if}\;8\leq y_{0}^{k}\leq y_{n+1}^{k}\leq 18\\ \sum_{k\in K}c_{w}\left(16-y_{0}^{k}\right)+c_{a}\left(y_{n+1}^{k}-16\right),\;\text{if}\;8\leq y_{0}^{k}\leq 18\leq y_{n+1}^{k} \end{array}\right.$$ (13)

Eq (8) represents each vehicle route that starts from the warehouse only once. Eq (9) implies that each vehicle should return to the same warehouse only once, and Eq (10) represents the vehicle that visits each customer exactly once. Constraint (11) states that balance should be maintained for the arrival and departure times of customer *i* for vehicle *k*. Constraint (12) indicates that the load of vehicle k cannot exceed its capacity. Eq (13) calculates the total wages of drivers under normal and abnormal work period conditions.

$$m_{k}\geq ts^{1},\;\forall k\in K$$ (14)

Constraint (14) ensures that the vehicle leaves the warehouse after or at *ts*^1^.

$$y_{n+1}^{k}\geq ts^{M+1},\;\forall k\in K$$ (15)

Constraint (15) ensures that vehicle returns to the warehouse before or at *ts*^*M*+1^.

$$y_{0}^{k}=m_{k},\;\forall k\in K$$ (16)

Constraint (16) states that the departure time of vehicle *k* from the warehouse is equal to the starting time of vehicle *k*.

## Algorithm analysis

The vehicle routing problem belongs to the NP-hard category, which is generally solved by some heuristic algorithms[25]. Among the heuristic algorithms based on the neighborhood search methods, the adaptive large neighborhood search (ALNS) has proven to be successful for a wide variety of vehicle routing problems [44–47]. The classical ALNS algorithm is an iterative process where, at each iteration, part of the current solution is destroyed and then reconstructed in the hope of finding a better solution [21,25]. Therefore, we apply an ALNS heuristic to solve the aforementioned optimization problem.

The main procedure is as follows. First, an initial solution is constructed to meet the demand of each customer. Second, the optimal route of each vehicle can be found by using the ALNS heuristic. Finally, the optimal departure time of each route is determined.

The step-by-step description of the algorithm is as follows:

**Step 1:** Use the greedy algorithm to construct an initial solution *S*^0^.
**Step 2:** Calculate the objective function value *obj*(*S*^0^). Set the best solution S* = *S*^0^ and best objective value *obj* = *obj*(*S*^0^).
**Step 3:** Let the initial temperature *t* = 100. The initial scores of removal heuristics *h*_*r*_ and insertion heuristics *h*_*i*_ are set to 0, and the weight of each heuristic *w*_*i*_ is initially set to 1.
**Step 4:** Destroy the incumbent solution *S*^*i*^ by using a removal heuristic *h*_*r*_.
**Step 5:** Construct a new solution *S*^*t*^ by using an insertion heuristic *h*_*i*_.
**Step 6:** Calculate the objective function value of the incumbent solution and mark it as *obj*(*S*^*t*^). If *obj* > *obj*(*S*^*t*^), then *obj* = *obj*(*S*^*t*^) and S* = *S*^*t*^; otherwise, the solution is accepted with a probability of exp(−(*obj*(*S*^*t*^)−*obj*)).
**Step 7:** Update the scores of removal and insertion heuristics and update the temperature *t* = *t* × *τ*.
**Step 8:** Check whether the end of the current segment has been reached. If the end has been reached, then proceed to Step 9; otherwise, proceed to Step 10.
**Step 9:** The weight of each heuristic *ρ*_*i*_ is updated and all scores are set to 0.
**Step 10:** Check whether the temperature or the time limit is reached. If one of the limits is reached, then proceed to Step 11; otherwise, proceed to Step 4.
**Step 11:** Search the best departure time for each route.
**Step 12:** Output the best solution S* (i.e., routes, departure time of each route, and corresponding objective value).

**Fig 3:**
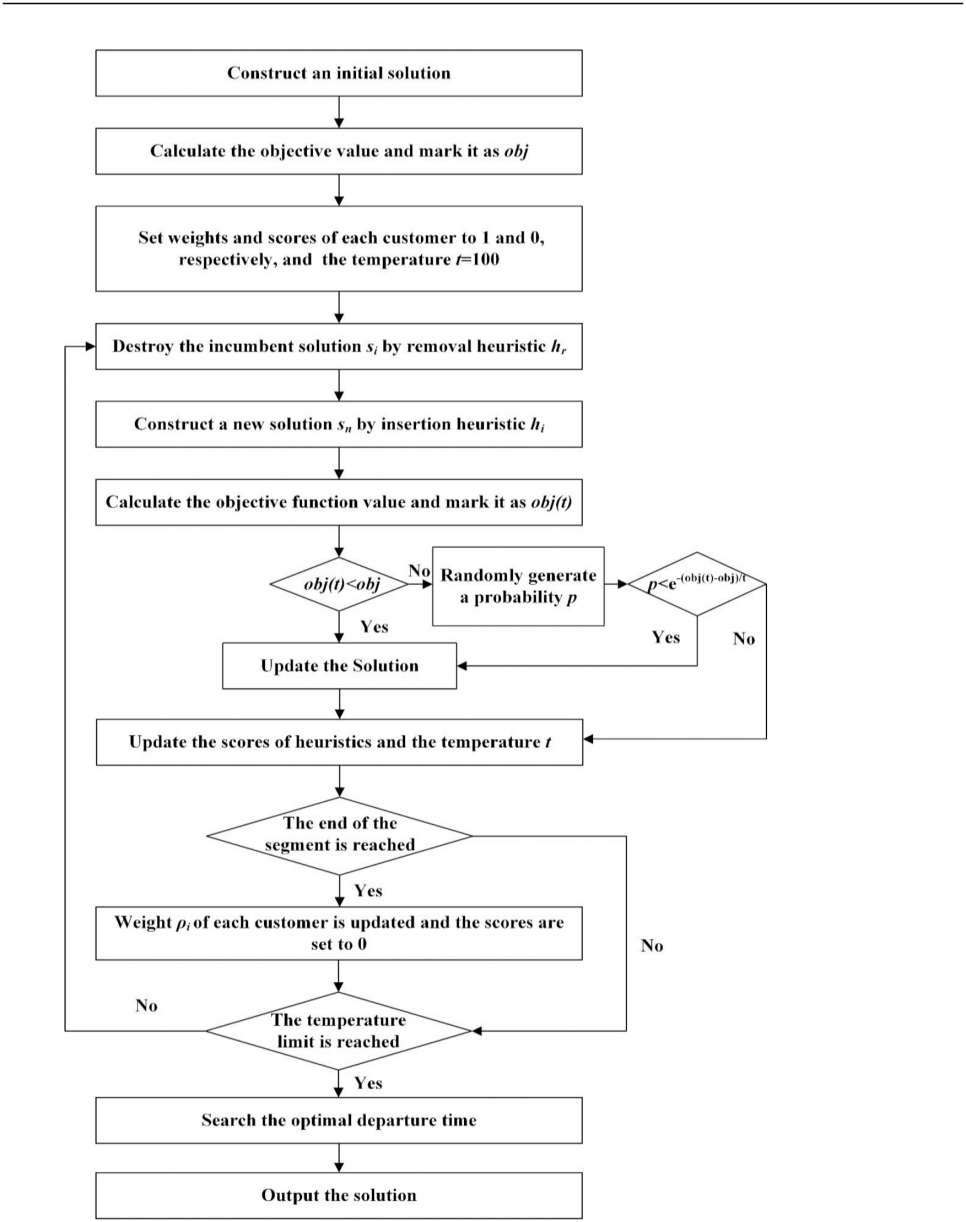
Flowchart of adaptive large neighborhood search algorithm.

Fig 3 shows the flow of adaptive large neighborhood search algorithm.

### Initial solution

We generate *M* routes first and insert all customers into these routes. Then, the initial solution is constructed. *M* is determined by

$$M=\left\lceil \frac{\sum_{i\in N_{0}^{\prime }}q_{i}}{mQ}\right\rceil ,$$ (17)

where *m* is the parameter that estimates the maximum vehicles needed. The initial solution is constructed using a basic greedy insertion heuristic. All customers are inserted into the routes based on their distances to the depot.

### Insertion and removal heuristics

We have developed two insertion and three removal heuristics in this study. These heuristics have been proven effective [35,48].

#### Insertion heuristics

1. Basic greedy insertion heuristic
  The basic greedy insertion heuristic inserts *μ* removed customers into the destroyed solution. Moreover, all feasible positions in the current route are tested and customers are inserted into positions with the lowest cost.
2. Revised greedy insertion heuristic
  The revised greedy insertion heuristic aims to improve the basic greedy insertion heuristic. The main difference is that, in each iteration, all the feasible insertion positions in every route are tested. The revised greedy insertion heuristic was used by Luo et al. [35]. It runs much slower than the basic greedy insertion heuristic.

#### Removal heuristics

Random, worst and neighborhood graph removal heuristics are used in this study. These heuristics are used to destroy the incumbent solution by removing *μ* customers.

1. Random removal heuristic
  The random removal heuristic randomly selects *μ* customers, which are removed from the incumbent solution.
2. Worst removal heuristic
  The worst removal heuristic selects *μ* customers that have the largest cost savings. The worst removal heuristic can improve the quality of the solution because the customers with large cost savings can be placed in better positions.
3. Neighborhood graph removal heuristic
  This heuristic aims to remove the most critical customers according to their historical information. The information is stored in a graph *G* = (*V*, *E*). The information stored in this graph is the weight *w*_*ij*_ of each edge (*i*, *j*)∈*E*. The weights are set to be infinite at the beginning and updated at each iteration and are calculated by:
  $$w_{ij}=\text{min}\left\{w_{ij},F_{ij}\left(w+f_{ij},\bar{v},d_{ij}\right)\times d_{ij}\right\}$$ (18)

Then, all *w*_*ij*_ for each customer $j,\in N_{0}^{\prime }$ are summed up. The *μ* customers to be removed are those with the highest $\sum_{j\in N_{0}}w_{ij}$. When a customer is removed, the surrounding customers are recalculated.

### Acceptance and stopping criteria

The acceptance criterion used in this article is adapted from the simulated annealing. The solution that is better than the current one is accepted directly, whereas the probability of accepting a worse solution is exp(−(*obj*(*S*^*t*^)−*obj*)), where *t* represents the current temperature.

Temperature *t* starts out at the initial temperature *T*_*Start*_. At each iteration, *t* is multiplied by cooling rate *c*, where 0 < *c* < 1. The iteration stops when either the temperature reaches the limit temperature or the limit on iteration times is reached. Both limits are fixed at the beginning.

### Large neighborhood

At each iteration, *μ* customers are selected from the incumbent solution to destroy the incumbent solution. *μ* is the parameter that controls the neighborhood size. With the increase of parameter *μ*, the neighborhood size becomes larger and more computational time is needed.

### Adaptive adjustment mechanism

Weight *ρ*_*i*_ is introduced to select the insertion and removal heuristics *h*_*i*_, *i*∈{1, 2,…,*k*} at each iteration. The idea is to update the score of each heuristic to measure its performance. A roulette wheel mechanism is adopted to choose the insertion and removal heuristics. The probability for heuristic *h*_*i*_ to be chosen is:

$$\rho_{i}/\sum_{j=0}^{k}\rho_{j},$$ (19)

The entire search process is divided into several segments. A segment consists of iterations of the ALNS heuristic. To start the entire search process, the score *π*_*i*_, *i*∈{1, 2,…,*k*} of each heuristic *h*_*i*_, *i*∈{1, 2,…,*k*} is set to 0. At the end of each iteration, all heuristics are adjusted based on their performances. The score (*π*_*i*_) is increased by *σ*_1_, *σ*_2_ or *σ*_3_, depending on the rule, which is presented in Table 1.

**Table 1:** Some adjustment parameters of score.

Parameter	Adjustment
*σ*_1_	The new solution is a globally best solution.
*σ*_2_	The new solution has not been visited and is better than the incumbent solution.
*σ*_3_	The new solution is accepted and has not been visited previously, but it is worse than the incumbent solution.

At the end of each segment, weight *ρ*_*i*_ is updated using the recorded score *π*_*i*_ as follows: *ρ*_*i*_ ≔ *αρ*_*i*_ + (1−*α*)×*π*_*i*_/*λ*_*i*_, where *α*∈[0, 1] is the controlling parameter and *λ*_*i*_ denotes the number of times heuristic *h*_*i*_ has been used in this segment. If *λ*_*i*_ equals to 0, then weight *ρ*_*i*_ is unchanged.

## Case study

### Data and parameter setting

**Fig 4:**
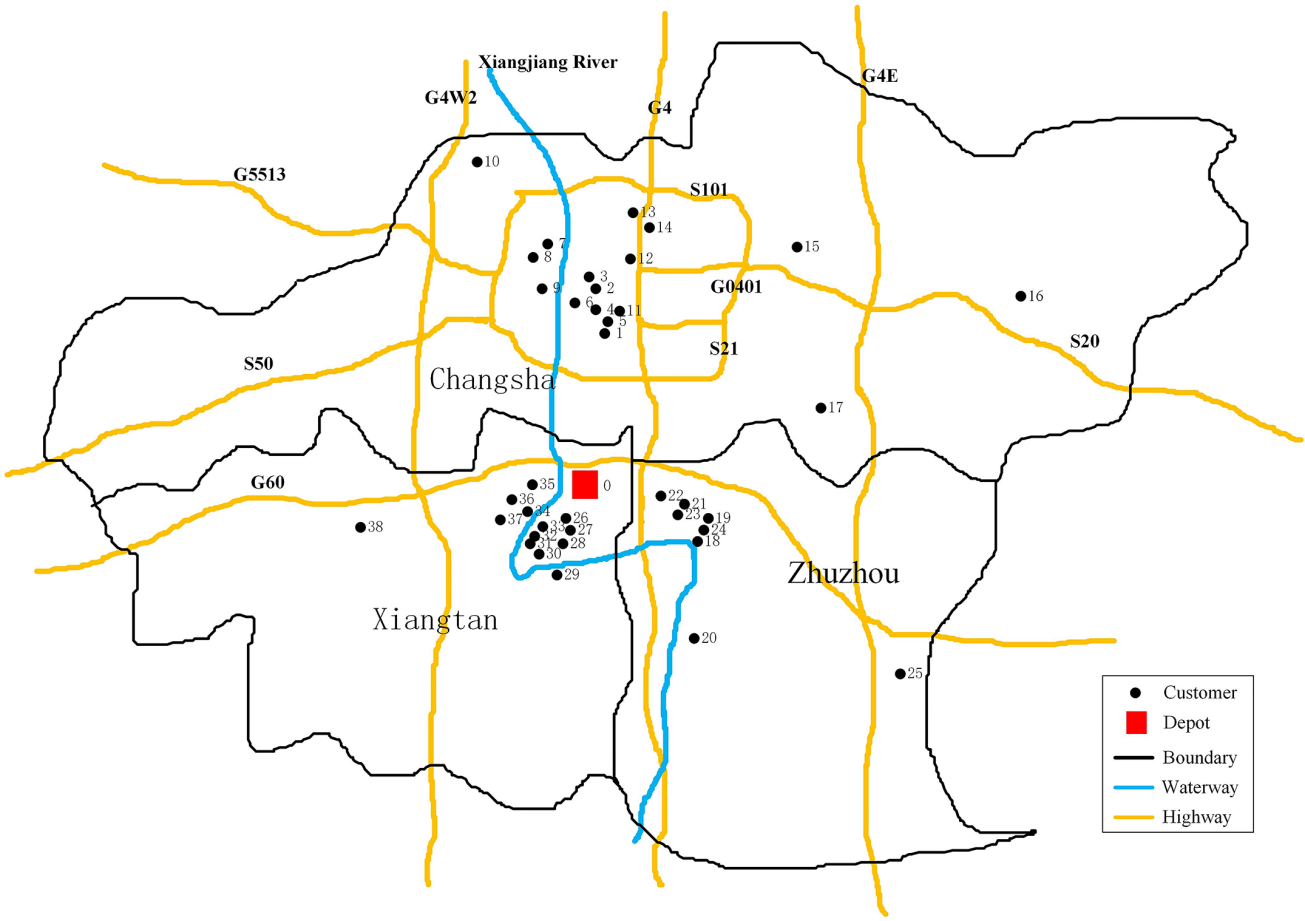
Distribution service network of a supermarket chain in Xiangtan, China.

A numerical example is used to illustrate the applications of the proposed model and the solution algorithm. The example distribution service network is a supermarket chain in Changsha, Zhuzhou and Xiangtan, Hunan province of China. The network consists of one depot and 38 customers, as shown in Fig 4.

All customers are served by the depot located in Xiangtan. The corresponding information on the locations, distance matrix, demands, and time windows of all the supermarket service nodes are shown in Appendices 1, 2, 3 and 4, respectively. The fixed costs of the vehicles, driver wages during normal work period, fuel cost, penalty cost of time-window violation, and CO_2_ emissions tax charge are set to 100 CNY per shift, 15 CNY per hour, 7.59 CNY per liter, 10 CNY per hour, and 80 CNY per ton, respectively[49][50]. The wages of drivers during the nonworking hours are twice that during the normal work period. The exchange rate of USD to CNY is 6.6533 (i.e. 1 USD = 6. 6533 CNY) [51].

In this case study, we consider the actual traffic congestion. The relationship between vehicle speed and time slot is shown in Table 2, and the driver wages during different periods are shown in Table 3.

**Table 2:** Average travel speed of vehicle at different time slots in Changsha.

Time slot	[0–5)	[5–7)	[7–9)	[9–12)	[12–14)	[14–16)	[16–19)	[19–21)	[21–24)
Speed (km/h)	40	35	22	30	25	35	22	32	40

(Sources: Data adapted from references [42,43])

**Table 3:** Different driver wages at different time slots.

Time slot	[0:00–8:00)	[8:00–18:00)	[18:00–24:00]
Wages(CNY/h)	30	15	30

The parameters of the ALNS algorithm are given as follows. According to the numerical experiment in the literature, the initial temperature *T*_*Start*_ is set to 100, the weight of each heuristic *ρ*_*i*_ is initially set to 1, whereas the scores of heuristics *π*_*i*_ are set to 0. The rest parameters are determined according to the computational experiments. The cooling rate c is 0.999; the score adjustment parameters are *σ*_1_ = 100, *σ*_2_ = 20, *σ*_3_ = 10; and the score adjustment controlling parameter is *α* = 0.5. After several preliminary experiments, the neighborhood size *μ* is fixed to 15. Moreover, the iteration stops when the temperature reaches the limit temperature or the limit on iteration times is set to 1000 s. These input data are considered the base case in the following analysis unless otherwise specified.

### Analysis and discussion of results

The proposed model and solution algorithm are applied to the sample supermarket chain distribution. The results are summarized as follows.

The proposed solution algorithm was coded in Java and run on a Dell N5040 laptop with a 2.13GHz Intel Pentium CPU and 4.00 GB RAM. The CPU time for searching the corresponding optimal solution is 37.7 seconds.

First, we address the effects of the different optimal objectives on the delivery routes and departure time. The following two schemes are employed: Scheme 1 aims to minimize the total of CO_2_ emissions, while Scheme 2 aims to minimize the total costs. According to the above algorithm, we obtain the corresponding solutions for the minimum total cost and minimum CO_2_ emissions, which are shown in Tables 4 and 5, respectively.

**Table 4:** Optimal routes to minimize the total costs (total costs = 3118.96 CNY).

Routes	Departure time
0-38-37-34-36-35-0	6:00
0-20-29-0	10:00
0-14-15-16-0	5:00
0-1-11-3-0	8:00
0-4-8-7-10-0	6:00
0-2-12-13-0	14:00
0-17-25-0	19:00
0-5-6-9-0	12:00
0-26-30-31-0	14:00
0-21-19-18-24-0	21:00
0-22-23-32-33-0	8:00
0-28-27-0	20:00

**Table 5:** Optimal routes to minimize the total emissions (total emissions = 354.65 kg).

Routes	Departure time
0-22-21-0	0:00
0-5-1-0	0:00
0-3-9-6-0	0:00
0-31-30-29-0	0:00
0-27-33-32-0	0:00
0-17-16-0	0:00
0-35-36-34-37-38-0	0:00
0-24-20-0	0:00
0-13-14-15-0	0:00
0-4-2-11-0	0:00
0-23-18-19-25-0	0:00
0-26-28-0	0:00
0-12-8-7-10-0	0:00

From Tables 4 and 5, we can deduce the following:

Minimal energy consumptions are not guaranteed for the vehicles along the shortest path. However, several common routes exist (i.e., the visiting sequences are the same with different optimization objectives). Furthermore, the distances between customers covered by the common routes are shorter and the loading rate in the routes is higher. Thus, they can be served by one vehicle with relatively less costs.

In Table 5, the optimal departure time for each vehicle to achieve minimal CO_2_ emissions is 0:00 midnight because the driving speed is at its optimum from 0:00–5:00.

To minimize the total carbon emissions with no departure time limitations, vehicles tend to depart from the depot with higher speeds and avoid the rush hours.

As shown in Table 6, the optimal departure time is 10:00, which considers the objective of minimizing the total cost of CO_2_ emissions and fuel consumption, whereas the departure time switch is 14:00, which considers the objective of minimizing the total costs. This is the result of the scheduling and routing schemes with the objective of optimizing the total cost that is related to CO_2_ emissions, in which more extra wages are paid because of the work during nonworking periods. In the scheme of optimizing the total cost, the departure time plan is chosen within the normal work period, and more costs related to CO_2_ emissions are incurred.

**Table 6:** Comparative analysis of the optimal departure time between two different schemes.

No.	Departure time	Scheme1-Total cost of CO_2_ Emissions and fuel consumption(CNY)	Scheme2-Total cost (CNY)
1	6:00	1104.255416	5337.601742
2	7:00	1092.808818	5029.613236
3	8:00	1106.667433	4688.701832
4	9:00	1076.540487	4576.30215
5	10:00	**1056.477081**	4459.950595
6	11:00	1096.634872	4445.17452
7	12:00	1095.192047	4519.835239
8	13:00	1058.688088	4471.238845
9	14:00	1072.191669	**4408.558375**
10	15:00	1110.497428	4664.553553
11	16:00	1098.897998	4977.733834
12	17:00	1116.788885	5324.513606

**Fig 5:**
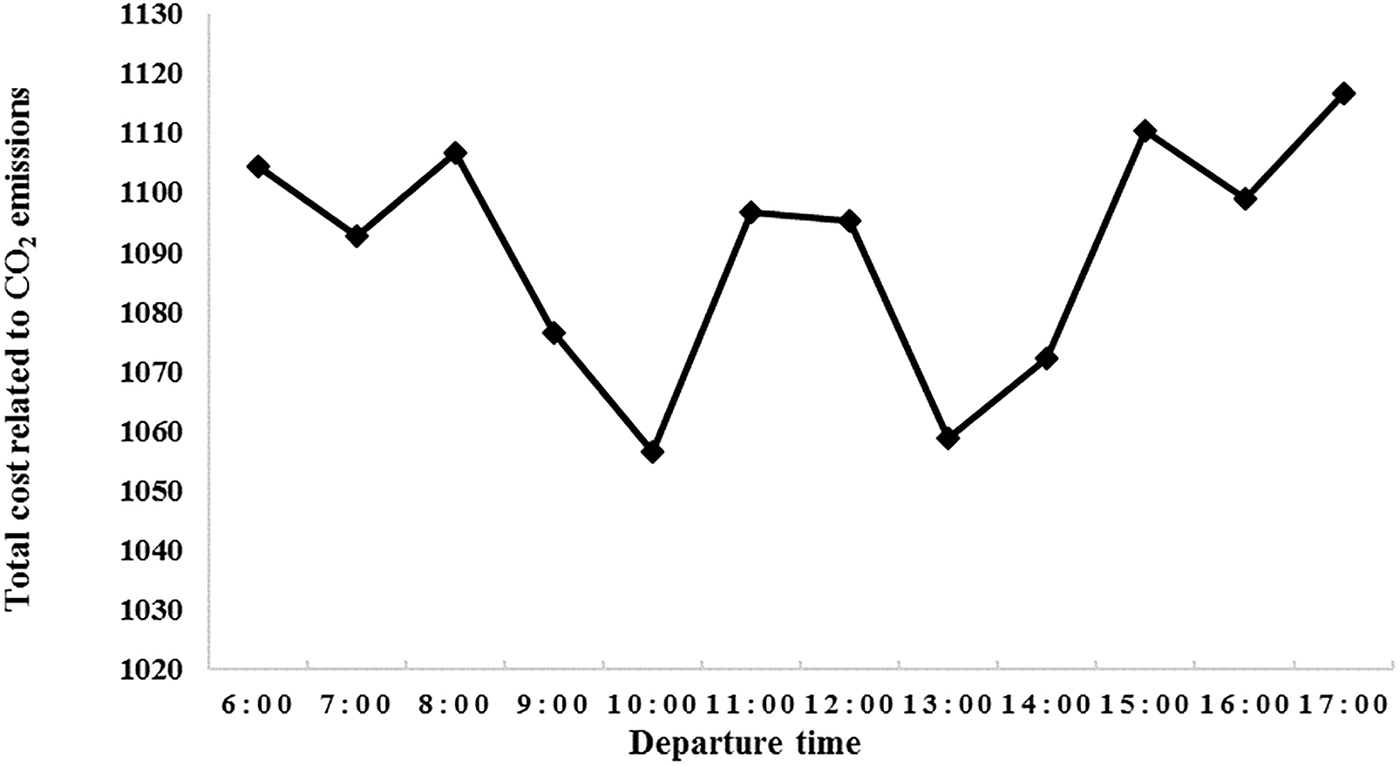
Relationship of CO_2_ emissions and departure time.

Fig 5 shows that the departure time slots of vehicles have significant effects on the total cost-related CO_2_ emissions. In this instance, the vehicle energy consumption at 10:00 a.m. is the least because the peak times (7:00 a.m.–9:00 a.m. and 12:00 p.m.–2:00 p.m., during which vehicles travel at a low speed), are avoided. Thus, the energy consumption is reduced. At 17:00 p.m., the energy consumption of the delivery vehicle is at the maximum because vehicles are caught up in the rush hour. At this time, the vehicle is extremely slow and its load remains relatively large for a longer period, thereby consuming a larger amount of energy.

CO_2_ emission in Scheme 1 is less than that in Scheme 2. The savings is up to 60.3118 kg, and the savings percentage is approximately 5.4%. This finding implies that scheduling the vehicle departure is an important method to reduce the total CO_2_ emissions of a delivery tour.

Next, the effects of different wages for overtime periods on delivery routing and departure time decisions are explored. We employ another two schemes: Scheme 3, in which the wages for different periods are the same, and Scheme 4, in which the wages for overtime periods are twice those of the wages for normal working periods.

Tables 7–9 show that although the driver wages per hour during overtime periods are doubled, the driver wages increase only by a percentage of 29.1%. The cost related to energy consumption and the penalty cost of time-window violation also increase. The optimal routes and the corresponding departure time should be determined based on the trade-off analysis between driver wages and other costs, especially the penalty cost of time-window violation. The vehicle routes and the corresponding departure time with different driver wages are different from those without extra wages. This finding implies that the extra driver wages affect routing and departure time decisions.

**Table 7:** The optimal routes and departure time without extra wages during nonworking periods.

Routes	Visiting sequences	Departure time
Route 1	0-24-20-0	13:00
Route 2	0-19-17-25-0	14:00
Route 3	0-38-37-34-36-35-0	20:00
Route 4	0-22-23-18-21-0	14:00
Route 5	0-14-15-16-0	5:00
Route 6	0-29-28-33-0	21:00
Route 7	0-9-8-7-0	5:00
Route 8	0-5-3-12-0	5:00
Route 9	0-4-11-13-10-0	5:00
Route 10	0-1-2-6-0	5:00
Route 11	0-26-27-0	19:00
Route 12	0-30-31-32-0	21:00

**Table 8:** The optimal routes and departure time with extra wages during nonworking periods.

Route	Visiting sequences	Departure time
Route 1	0-38-37-34-36-0	6:00
Route 2	0-20-29-0	10:00
Route 3	0-14-15-16-0	5:00
Route 4	0-1-11-3-0	8:00
Route 5	0-4-8-7-10-0	6:00
Route 6	0-2-12-13-0	14:00
Route 7	0-17-25-0	19:00
Route 8	0-5-6-9-0	12:00
Route 9	0-26-30-31-0	14:00
Route 10	0-21-19-18-24-0	21:00
Route 11	0-22-23-32-33-0	8:00
Route 12	0-28-27-0	20:00

**Table 9:** Comparative analysis of system performances with extra wages during overtime periods.

	Scheme 3 (without extra wages)	Scheme 4 (with extra wages)
Total cost	2878.94	3118.96
Energy consumption and CO_2_ emissions	1077.40	1086.19
Driver wages	532.46	687.61
Time-window violation costs	69.08	145.16
Fixed cost	1200.00	1200.00

### Practice insights

Based on the above analysis, we have obtained some practice insights shown as follows. There are several key results of this research that have direct managerial insights for delivery practice. Results show that: (1) the shortest routes based on the minimal total costs are different from those routes based on minimal total CO_2_ emissions, (2) the choice of starting time has a significant influence on the overall energy consumption and CO_2_ emissions, and (3) the extra driver wages affect the routing and departure time decisions. In this study, the optimal travel time reduces the CO_2_ emissions by almost 5.4% compared with the worst travel time. Thus, considering the road traffic congestion factors and the reasonable arrangements for the departure time of vehicles is an important way to reduce logistics costs and CO_2_ emissions.

## Conclusions

This paper investigated the joint optimization problem of the green vehicle scheduling and routing problem, taking time-varying vehicles speeds and different wages in different time slots into considerations. A corresponding joint optimization model of the departure time schedule of vehicles and the routing problem with time-varying speeds is presented. The objective of the proposed model not only aims at minimizing transportation related costs (i.e. fuel consumptions and drivers wages) and fixed cost of vehicles, but also considers the cost of carbon emission. Based on the characteristics of the optimization model, a heuristic algorithm based on the adaptive large neighborhood search algorithm is provided to solve the problem. The optimal model and corresponding algorithms were evaluated by a real-life case study on the joint vehicle scheduling and routing problem. The effects of the different optimal objectives on the delivery routes and departure time are also analyzed.

Meanwhile, the following new insights and important findings are obtained from this study. First, the choice of starting time has a significant influence on the overall energy consumption and CO_2_ emissions. Second, the extra driver wages affect the routing and departure time decisions. Third, it is a significant way for logistics delivery operators to reduce logistics costs and CO_2_ emissions by joint optimization of the departure time of vehicles and routing considering the road traffic congestion factors.

Although the numerical results presented in this study can be explained logically, larger and realistic cases are necessary to further justify the findings using the proposed model. It is also important to address the robust green vehicle routing problem under stochastic traffic conditions.

## Supporting information

S1 Appendix Details of customers’ names and their numbers. (DOCX)

S2 Appendix Details of distance matrix of depot and customers. (XLSX)

S3 Appendix Details of demand of customers. (XLSX)

S4 Appendix Details of service time windows of customers. (XLSX)
