# Supplementary material for: Joint optimization of green vehicle scheduling and routing problem with time-varying speeds
Source: PLoS One. 2018 Feb 21;13(2):e0192000. doi: 10.1371/journal.pone.0192000 (PMC5821442; doi:10.1371/journal.pone.0192000)
Supplement: S1 Appendix — (DOCX) [file pone.0192000.s001.docx]

**Appendix-1 Customers’ names and the corresponding number**

| Customer name | Customer No. |
| --- | --- |
| Hongxing | 1 |
| Dongtang | 2 |
| Ziyuan road | 3 |
| University of foestry | 4 |
| Jinggui road | 5 |
| Nanguo | 6 |
| Jinxing road | 7 |
| West station | 8 |
| Wangjiawan | 9 |
| Wangcheng | 10 |
| Xiledi | 11 |
| East station | 12 |
| Xingsha | 13 |
| Kaiyuan | 14 |
| Yongan | 15 |
| Lihua road | 16 |
| Zhentou | 17 |
| Guoan | 18 |
| Dongdu | 19 |
| Lukou | 20 |
| Tianxin | 21 |
| Xiangtian bridge | 22 |
| Shanmutang | 23 |
| Fuhua | 24 |
| Cicheng | 25 |
| Bantang | 26 |
| Jinqiao | 27 |
| Dongfanghong | 28 |
| Yisu river | 29 |
| Yuetang | 30 |
| Gangcheng | 31 |
| Liancheng | 32 |
| Baota | 33 |
| Baishi | 34 |
| University of science | 35 |
| Guangyun | 36 |
| Jinhai | 37 |
| Jiangnan | 38 |
